# Chrono-Nutrition Has Potential in Preventing Age-Related Muscle Loss and Dysfunction

**DOI:** 10.3389/fnins.2021.659883

**Published:** 2021-04-16

**Authors:** Shinya Aoyama, Yasukazu Nakahata, Kazuyuki Shinohara

**Affiliations:** Graduate School of Biomedical Sciences, Nagasaki University, Nagasaki, Japan

**Keywords:** circadian rhythm, sarcopenia, nutrition, aging, muscle, chrono-nutrition

## Abstract

The mammalian circadian clock systems regulate the day–night variation of several physiological functions such as the sleep/wake cycle and core body temperature. Disturbance in the circadian clock due to shiftwork and chronic jetlag is related to the risk of several disorders such as metabolic syndrome and cancer. Recently, it has been thought that shiftwork increases the risk of sarcopenia which is characterized by age-related decline of muscle mass and its dysfunctions including muscle strength and/or physical performance. First, we summarize the association between circadian rhythm and the occurrence of sarcopenia and discuss its mechanistic insight by focusing on the muscle function and molecular clock gene in knockout or mutant mice. The clock gene knockout or mutant mice showed early aging phenotypes, including low survival rate and muscle loss. It suggests that improvement in the disturbance of the circadian clock plays an important role in the aging process of healthy muscles. Nutritional intake has the potential to augment muscle growth and entrain the peripheral clock. Second, we discuss the potential of chrono-nutrition in preventing aging-related muscle loss and dysfunction. We also focus on the effects of time-restricted feeding (TRF) and the distribution of protein intake across three meals.

## Introduction

Several physiological functions such as sleep/wake cycle exhibit day–night variation. Most of the physiological events based on day–night variations are regulated by the circadian clock systems consisting of transcriptional and translational negative feedback loop of core clock genes. The heterodimer of brain and muscle ARNT-like 1 (BMAL1) and circadian locomotor output cycle kaput (CLOCK) induces the transcription of *Period1/2* (*Per1/2*) and *Cryptochrome1/2* (*Cry1/2*) by binding to E-box-binding site within promoter regions of *Per1/2* and *Cry1/2* (Gekakis et al., 1998). After their transcription and translation, PER1/2 and CRY1/2 inhibits its own transcription mediated by the BMAL1 and CLOCK complex. PER1/2 and CRY1/2 are also degraded by post-translational modifications such as phosphorylation and ubiquitination (Busino et al., 2007). However, the transcriptions of *Bmal1* and *Clock* are suppressed and activated by *nuclear receptor subfamily 1, group D* (*Rev-erbα*), and *RAR-related orphan receptor* (*Rors*), respectively, by binding to the ROR-responsive element (Preitner et al., 2002; Sato et al., 2004). The transcriptions of *Rev-erbα* and *Rors* are regulated by binding to the E-box-binding elements of the BMAL1 and CLOCK complex (Preitner et al., 2002; Sato et al., 2004). The clock genes are expressed in the whole body, and the negative feedback loop in several tissues regulates the tissue-dependent day–night variations of physiological functions (Zhang et al., 2014). The mammalian circadian clock system is divided into two layers, namely, the central clock in the suprachiasmatic nucleus (SCN) of the hypothalamus and peripheral clock in the peripheral tissue, including brain areas other than the SCN, liver, skeletal muscle, and so on. Light stimulates SCN neuronal activity, which acts as the main entrainer of the central clock in the SCN. The central clock synchronizes the peripheral clocks through the neural and endocrine signaling pathways such as the sympathetic nervous system and glucocorticoid signaling (Schibler et al., 2003; Shibata, 2004). The peripheral clocks are entrained not only by stimulus from the central clock but also by other factors such as a meal, exercise, and stress in a central clock-independent manner (Tahara and Shibata, 2013). Although exercise, insulin, glucocorticoid and hypoxia entrain the muscle clock, the muscle clock is less susceptible to feeding-dependent entrainment than the liver clock (Harfmann et al., 2015; Oishi et al., 2017; Peek et al., 2017). Such rhythmic regulation changes with life stages. In the older adults, the amplitude of many physiological rhythms such as waking activity dampens and some of them exhibit phase shifts (Hood and Amir, 2017). The analysis of clock gene knockout mice suggests that aging is accelerated due to the circadian clock disturbance (Kondratov et al., 2006). Sarcopenia, which is one of the common diseases among older adults, is muscle dysfunctions due to aging. In recently, some studies show the disturbance of sleep and circadian rhythms relates to the risk of sarcopenia (Piovezan et al., 2015; Choi et al., 2020). Herein, we discuss the following two topics: (1) the association between circadian rhythm and age-related muscle loss and dysfunction in human which are diurnal (day active) and discuss mechanistic insights focusing on the molecular clock gene using knockout or mutant mice models which are nocturnal animal (night active), and (2) the potential of chrono-nutrition in preventing age-related muscle loss and dysfunction.

## Circadian Rhythm and Sarcopenia

### Human Study

Sarcopenia is characterized by age-related muscle loss and its dysfunctions such as strength and/or physical performance (Chen et al., 2014). Its pathogenesis is complex and includes multiple factors such as age-related decline of hormonal and metabolic system, malnutrition and so on (Chen et al., 2014). Recently, it is reported that sleep is also play a partial role in the aging of healthy skeletal muscle, such as sarcopenia and metabolic dysfunction. Longer sleep duration (>9 h) leads to a higher risk of sarcopenia than optimal sleep duration (approximately 7 h) (Kwon et al., 2017). A similar association was observed in postmenopausal women (Fex et al., 2012). Another study showed that the prevalence of sarcopenia in older adults is higher with shorter sleep duration (<6 h) and longer (>8 h) sleep duration (Hu et al., 2017; Rubio-Arias et al., 2019). A U-shaped relationship between sleep duration and the prevalence of sarcopenia was observed. These associations between sleep duration and the prevalence of sarcopenia remained after adjusting for confounding factors including age, BMI, physical activity, smoking, energy intake and so on (Hu et al., 2017; Kwon et al., 2017). Although mechanistic insight of association between sleep duration and sarcopenia remains unclear, insulin resistance, chronic inflammation and anabolic hormone could be possible link between sleep duration and sarcopenia (Patel et al., 2009; Piovezan et al., 2015; Smiley et al., 2019). In addition to sleep duration, sleep quality, assessed using the Pittsburgh Sleep Quality Index (PSQI), was also associated with sarcopenia (Buchmann et al., 2016; Lucassen et al., 2017). As with the relationship between sleep duration and sarcopenia, the PSQI score was still associated with muscle mass after adjusting for age, physical activity, HOMA-IR and testosterone (Buchmann et al., 2016). Frailty is an age-related clinical state that indicates a decline in physiological function and enhanced vulnerability to stressors. Physical functions such as grip strength and gait speed are also included in the sarcopenia diagnostic criteria. Similar to the relationship between sleep duration and sarcopenia, shorter (<6 h) and longer (>8 h) sleep duration significantly increased the risk of frailty (Pourmotabbed et al., 2020). Additionally, the risk of frailty is increased by other sleep parameters such as daytime sleepiness, sleep-disordered breathing, and prolonged sleep latency (Endeshaw et al., 2009; Ensrud et al., 2009, 2012; Vaz Fragoso et al., 2009; Nakakubo et al., 2019). These data suggest that adequate sleep might have preventive effects on the development of sarcopenia. Chronotype is also related to the prevalence of sarcopenia. Yu et al. reported that the evening chronotype was associated with an increased risk of sarcopenia in older men, independent of sleep duration (Yu et al., 2015). Recently, an analysis of the data of 9105 Korean people including non-shift workers and shift workers reported that shift workers had a higher risk of sarcopenia (Choi et al., 2019). Further analysis showed that a shift worker with an irregular schedule had the highest risk of sarcopenia (Choi et al., 2019). These data suggest that inadequate sleep and disturbance of circadian clocks were related with the risk of sarcopenia.

### Animal Study

Although human studies have suggested that low sleep quality and shiftwork, which cause disturbance of the circadian rhythm, are risk factors for sarcopenia, the underlying mechanism is not yet fully understood. Evidence from animal studies may support the mechanistic insight connecting the relationship between disturbance of the circadian clock and occurrence of sarcopenia (Chatterjee and Ma, 2016; Aoyama and Shibata, 2017). Whole-body *Bmal1*-knockout mice exhibited early age-related muscle loss (Kondratov et al., 2006). Similar findings were observed in *Clock mutant* mice and *Rev-erbα* knockout mice (Andrews et al., 2010; Mayeuf-Louchart et al., 2017). It was expected that the muscle clock was contributed to muscle volume from the results of the restored body weight loss in muscle-specific *Bmal1* rescue in whole-body *Bmal1* knockout mice (muscle mass was not evaluated) (McDearmon et al., 2006). However now it is thought that its contribution to age-related muscle loss is small because muscle loss was not observed in muscle-specific *Bmal1*-knockout mice in contrast to whole-body *Bmal1*-knockout mice (Dyar et al., 2014; Schroder et al., 2015). In the discrepancy of phenotype between muscle-specific *Bmal1* rescued mice and muscle-specific *Bmal1* knockout mice, prevention of body weight loss by muscle-specific *Bmal1* rescue may be explained by restoration of locomotor activity (McDearmon et al., 2006). Although the muscle-specific *Bmal1* knockout mice showed the normal locomotor activity (Hodge et al., 2015), they also had gait issues similar to that observed in aging humans (Schroder et al., 2015). The broader impacts of intrinsic muscle clock on musculoskeletal system might contribute to in turn locomotor activity through improvement of gait. In addition to muscle-specific effects, it is suggested that the timing of *Bmal1* expression is key to development aging. In tamoxifen-inducible whole-body *Bmal1-*knockout mice, the deletion of *Bmal1* after muscle development (>3 months old) did not affect body weight loss and some early aging phenotypes such as glucose intolerance (Yang et al., 2016). These data suggest that *Bmal1* expression during development is important for maintaining muscle volume. On the other hand, the muscle-specific *Bmal1*-knockout mice also exhibited lower muscle strength, a shift to oxidative fiber type and muscle fibrosis (Dyar et al., 2014; Schroder et al., 2015). It suggests that the intrinsic muscle molecular clock affects muscle quality rather than muscle mass. Skeletal muscle has a key role of whole-body energy metabolism (Zurlo et al., 1990). Intrinsic muscle clock controls the day-night variations of glucose, amino acids and lipid metabolism, and muscle-specific *Bmal1* knockout mice show the glucose intolerance and insulin resistance (Dyar et al., 2014, 2018; Harfmann et al., 2016). Considering that insulin resistance and diabetes are one of the risk factors of sarcopenia, the metabolic dysfunctions due to disturbance of muscle clock may encourage the development of sarcopenic phenotypes such as a decline of muscle strength. Taken together, the muscle clock affects the quality of skeletal muscle such as a shift of muscle fiber type and muscle strength while the molecular clock in non-muscle fiber cell such as myogenic progenitors may contribute to age-related muscle decline shown in whole-body *Bmal1* knockout mice.

## The Role of Molecular Clock on Muscle Growth and Myogenesis

### Muscle Protein Synthesis and Degradation

The balance between muscular protein synthesis and degradation is important for the regulation of skeletal muscle mass and strength. *Atrogin1* (*F-box protein 32*) and *Muscle RING-finger protein-1* (*Murf1*) are rhythmic genes found in the circadian transcriptome (McCarthy et al., 2007; Miller et al., 2007; Dyar et al., 2015). These genes are E3 ubiquitin ligase and the key genes in the progression of muscle atrophy, and each knockout mouse shows resistance to denervated muscle atrophy (Bodine et al., 2001). The day–night variation of *Atrogin1* was also observed in the hindlimb unloading-induced muscle atrophy model but not in hindlimb unloaded *Clock mutant* mice (Aoyama et al., 2018). Dyar et al. reported that *Murf1* transcription was downregulated by *Rev-erbα* and its expression was found to be reduced due to the rescue of *Rev-erbα* in muscle-specific *Bmal1*-knockout mice (Dyar et al., 2018). These data suggest that a circadian clock is associated with muscle atrophy. Muscle protein synthesis is important for muscle growth and repair after exercise-induced muscle hypertrophy. The mTOR pathway is the key signaling pathway in protein synthesis, and this pathway is activated by resistance exercise training and nutrition such as amino acids (Stokes et al., 2018). Lipton et al. reported that phosphorylation of BMAL1 by S6K (ribosomal S6 protein kinase), which is a substrate of mTOR, regulates the day–night variation of translation in the liver (Lipton et al., 2015). The results of circadian transcriptomics and metabolomics of muscle-specific *Bmal1*-knockout mice revealed that amino acid metabolism was dramatically reprogrammed by the loss of muscle *Bmal1* (Dyar et al., 2018). Indeed, the translation level, which was assessed with puromycin-labeled peptides, was found to be altered in the muscle-specific *Bmal1*-knockout mice (Dyar et al., 2018). These results indicate that the circadian clock controls the day–night variation in muscle protein synthesis. It is possible that the day–night variation in muscle protein synthesis depends on the fasting/feeding cycle and locomotor activity rhythm rather than the direct regulation of the muscle clock because muscle protein synthesis shows a higher response to feeding and physical activity. In recent years, Kelu et al. (2020) reported that muscle exhibited day–night differences in growth independent of physical activity and feeding in zebrafish which was diurnal. Muscle anabolism is activated during the day, while the muscle catabolism is high during the night. Such day–night variation remained in the inactive muscle and under the no-feeding condition. However, the day–night variation in muscle growth disappeared due to clock disruption. Taken together, the circadian clock regulates the day–night variation in muscle growth, and this is augmented by locomotor activity rhythm and the feeding/fasting cycle.

### Myogenesis

**FIGURE 1:**
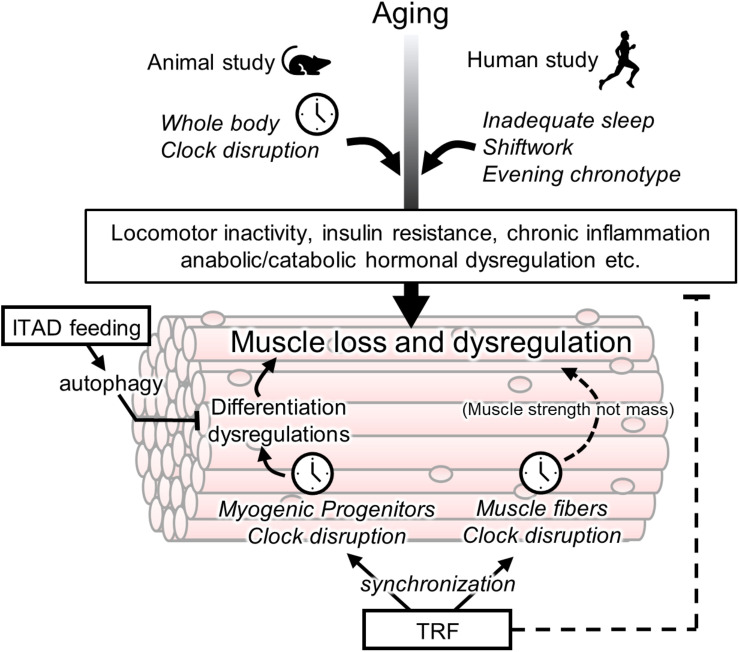
Association between circadian clock and age-related muscle loss and targets of chrono-nutrition. Age-related muscle loss is accelerated by a disturbance of a circadian clock in the animal and human studies. Its disturbance may be involved in early onset of sarcopenia via several non-muscular rhythmic functions such as a locomotor activity and insulin resistance. The muscle fiber clock has a impact on the age-related weakness of muscle strength. The circadian clock in myogenic progenitors controls muscle differentiation however its involvement in sarcopenia is unclear yet. The isocaloric twice-a-day (ITAD) feeding promotes the myogenesis via the activation of autophagy in myogenic progenitor cells. The time-restricted feeding (TRF) may have a potential for prevention of age-related muscle dysfunctions via entrainment of the peripheral clock including muscle fiber and progenitor cells. In addition to the role of TRF as an entrainer, TRF also maintains the daily homeostasis of metabolism such as prevention of insulin resistance.

Myofilament architecture was disrupted in the skeletal muscle of *Bmal1*-knockout mice and *Clock mutant* mice (Andrews et al., 2010). *Myod* is one of the key genes participating in muscle myogenesis, the process of myotube formation from satellite cells and myoblast cells. The day–night variation in *Myod* was observed in the skeletal muscle of wild-type mice but not in *Clock mutant* mice (Andrews et al., 2010). Its rhythmic expression is regulated by the BMAL1 and CLOCK complex (Andrews et al., 2010). Chatterjee et al. (2013, 2015) reported the role of *Bmal1* in myogenesis. Suppressed expression of myogenic genes such as *Myod*, *Myog*, and *Myf5* and impairment of myogenesis were observed in the myoblasts of *Bmal1*-knockout mice (Chatterjee et al., 2013). In addition, the authors reported the *in vivo* function of *Bmal1* in skeletal muscle regeneration (Chatterjee et al., 2015). Freeze- or cardiotoxin-induced muscle regeneration was suppressed by the depletion of *Bmal1* (Chatterjee et al., 2015). Recently, there has been evidence for the potential of *Rev-erbα* in preventing myogenesis through augmented satellite cell expansion and myogenic progression (Chatterjee et al., 2019). In addition, CRY1 and CRY2 regulate the proliferation and differentiation of muscle satellite cells negatively and positively, respectively. The CRY2-dependent acceleration of muscle cell differentiation was controlled by the rhythmic expression of *CyclinD1* and *Tmem176b* due to binding to BCLAF1 (Lowe et al., 2018). These results indicate that the myogenic process is regulated by the circadian clock (Figure 1). In recent study, MYOD1 regulates not only the myogenesis but also the amplitude of *Bmal1* and clock-controlled genes such as *Tcap* (Hodge et al., 2019). Considering the role of intrinsic muscle clock in muscle strength and metabolism, *Myod1* is one of therapeutic target as a daily maintenance of skeletal muscle functions. Recently, in satellite cells, aging reprogrammed the expression profile of rhythmic genes without any change in clock gene expression (Solanas et al., 2017). Although the physiological effects caused by aging-related reprogramming are fully unclear, these data may help to understand the mechanism underlying the relationship between the occurrence of sarcopenia and disturbance in the circadian rhythm.

## Therapeutic Potential of Chrono-Nutrition Against Are-Related Muscle Dysfunctions

Time-restricted feeding (TRF), which is restricted only to the eating time window but not calorie restriction, has strong preventive or therapeutic potential for metabolic dysfunction such as obesity and insulin resistance (Longo and Panda, 2016; Manoogian and Panda, 2017; Chaix et al., 2019). In *Drosophila*, TRF improves obesity-induced muscle dysfunction, including sarcomere disorganization, mitochondrial function, and insulin resistance (Villanueva et al., 2019) (Figure 1). In that study, TRF was also effective in the constant light-induced muscle dysfunction (Villanueva et al., 2019). TRF also attenuated age-related decline of cardiac muscle function in *Drosophila* (Manoogian and Panda, 2017). Martinez-Lopez et al. reported the beneficial effects of an isocaloric twice-a-day (ITAD) feeding model, which has two eating windows (Martinez-Lopez et al., 2017). ITAD feeding increased type IIB fibers, which are glycolytic muscle fibers (Martinez-Lopez et al., 2017). Moreover, an increase in myogenic genes was observed in the ITAD-fed mice, suggesting that ITAD feeding enhanced myogenesis of glycolytic muscle fibers. In addition, ITAD feeding prevented age-related metabolic defects such as glucose intolerance and mitochondrial dysfunction (Martinez-Lopez et al., 2017). These beneficial effects of ITAD feeding were not observed in myogenic progenitor-specific *Atg7*-knockout mice (Martinez-Lopez et al., 2017). Autophagy of myogenic progenitor cells is required for glycolytic muscle fiber expansion due to ITAD feeding (Figure 1). These data suggest that intermittent fasting-induced autophagy within a day is one of the key beneficial effects of time-controlled feeding such as TRF and ITAD feeding. Other animal studies have shown a difference between the TRF conducted during the active phase (night time) and that conducted during the rest phase (day time) (Abe et al., 2019; Aoyama et al., 2019). The TRF conducted during the active phase has beneficial effects on muscle growth and muscular protein synthesis compared with that conducted during the rest phase (Abe et al., 2019; Aoyama et al., 2019). Taken together, considering that muscle loss is particularly observed in glycolytic muscle fibers of patients with sarcopenia, ITAD feeding during the active phase may be an effective nutritional intervention for the prevention of sarcopenia.

A few human studies have shown the effects of TRF on skeletal muscle function in young and older adults. The TRF induced between 0800 and 1400 improved the 24-h glucose levels and increased the expression of the anti-aging gene *SIRT1* and the autophagy gene *LC3A* in the blood compared with the TRF induced between 0800 and 2000 (Jamshed et al., 2019). In terms of skeletal muscle function, 2 weeks of TRF induced between 0800 and 1600 improves insulin and anabolic responses in the skeletal muscle of healthy young men (Jones et al., 2020). Indeed, TRF increased the skeletal muscle uptake of glucose and branched-chain amino acids (BCAAs) (Jones et al., 2020). The effects of TRF in men who are overweight/obese have been elucidated using the circadian transcriptome of skeletal muscle (Lundell et al., 2020). TRF conducted for 8 h stimulated rhythmicity of amino acid metabolites and its transporter expression without perturbing the expression of molecular clock genes (Lundell et al., 2020). Additionally, considering that the transcriptomics and metabolomics data from human skeletal muscle after acute sleep loss suggest the muscle degradation according to the change of circadian clock expression (Cedernaes et al., 2018), synchronization of the muscle clock by TRF may suppress muscle degradation due to sleep loss. In older overweight adults, a 4-week TRF intervention resulted in body weight loss, although improvements in cognitive and physical function were not observed in this pilot study (Anton et al., 2019). Although few studies have reported about the effects of TRF on muscle function in older adults, evidence of the TRF effects focusing on skeletal muscle function in human studies is increasing.

Protein intake is important for maintaining balance between muscle protein synthesis and protein degradation. Dietary protein and amino acids act not only as a source of body protein but also as activators of mTOR signaling. Compared with young adults, a higher amount of dietary protein was required in older adults for a greater response to muscle protein synthesis (Fujita and Volpi, 2006), suggesting that the sensitivity of amino acids in the skeletal muscle decreased with aging. Considering the reduction in meal size in elderly people, we need ingenuity to take enough amount of protein across the three meals. A diet survey conducted in several countries showed that protein intake at breakfast was at a low level, and the daily distribution of protein was skewed (National Center for Health Statistics About the National Health and Nutrition Exmaination Survey (NHANES), 2012; Tieland et al., 2015; Ishikawa-Takata and Takimoto, 2018). Some human and animal studies have shown that even distribution of dietary protein across the three meals increased muscle protein synthesis and muscle mass compared with the skewed distribution, which indicates a low protein meal at breakfast (Mamerow et al., 2014; Norton L.E. et al., 2016). Another report showed that protein supplementation at breakfast and lunch for 24 weeks increased whole-body lean tissue mass in healthy older adults (Norton C. et al., 2016). The distribution of dietary protein is associated with muscle strength and frailty (Bollwein et al., 2013; Farsijani et al., 2017). In addition to muscle function, dietary protein has the potential to entrain peripheral clocks through the regulation of insulin growth factor-1 and glucagon signaling in an animal study (Ikeda et al., 2018). Tahara et al. (2017) reported age-related changes in peripheral clock entrainment according to feeding, and aged mice were susceptible to the feeding-induced phase shift of peripheral clocks such as the kidney and submandibular gland. In other words, a high protein meal at breakfast may have two effects on the prevention of sarcopenia: (1) augmentation of muscle growth and (2) improvement of the disrupted circadian clock through entrainment of peripheral clocks.

## Summary and Perspectives

In this review, we confirmed that inadequate sleep and shiftwork, which disturbs the circadian clock, were risk factors for sarcopenia and frailty. Considering that age-related muscle loss is observed in the whole body but not muscle-specific disruption of the molecular clock, it is suggested that the risk of sarcopenia may be increased due to the disturbance of the circadian clock in non-muscle tissues such as the SCN or myogenic progenitor cells. On the other hand, the broader impact of muscle clock on the musculoskeletal system such as muscle strength and gait might also have a role in preventing a sarcopenia as these changes of musculoskeletal system might precede the loss of muscle mass. Further studies are warranted to gain a clearer understanding of the mechanism underlying the interaction between circadian clock disturbance and age-related muscle dysfunction. We also discussed the potential of chrono-nutrition in preventing muscle aging in terms of two aspects: (1) to consider adequate timing or distribution of protein intake in a day and (2) to reset the peripheral clock. Although there is evidence for the beneficial effect of scheduled controlled feeding such as TRF in human and animal studies, the role of a circadian clock in the effects of chrono-nutrition on muscle health is not fully understood. Further research is needed to reveal the mechanism and elucidate the anti-aging effects of chrono-nutrition in older subjects who experience disturbance in the circadian rhythm, such as shift workers.

## Author Contributions

SA was involved in conceptualizing and writing the manuscript. KS and YN were involved in conceptualizing and editing the manuscript. All authors contributed to the article and approved the submitted version.

## Conflict of Interest

The authors declare that the research was conducted in the absence of any commercial or financial relationships that could be construed as a potential conflict of interest.
